# A unilateral whiteout lung in child with multisystem inflammatory syndrome associated with COVID-19 due to SARS-CoV-2: one case report of a boy

**DOI:** 10.1186/s12890-023-02428-1

**Published:** 2023-05-04

**Authors:** Tidarat Sriboonyong, Poomiporn Katanyuwong, Jarin Vaewpanich

**Affiliations:** 1 Division of Pediatric Pulmonology, Department of Pediatrics, Faculty of Medicine Ramathibodi Hospital, Mahidol University, Bangkok, Thailand; 2 Division of Pediatric Cardiology, Department of Pediatrics, Faculty of Medicine Ramathibodi Hospital, Mahidol University, Bangkok, Thailand; 3 Division of Pediatric Critical Care Medicine, Department of Pediatrics, Faculty of Medicine Ramathibodi Hospital, Mahidol University, Bangkok, Thailand; 4 Department of Pediatrics, Faculty of Medicine Ramathibodi Hospital, Mahidol University, Bangkok, 10400 Thailand

**Keywords:** Unilateral whiteout lung, Multisystem inflammatory syndrome in children, Chest radiograph

## Abstract

**Background:**

Multisystem inflammatory syndrome in children (MIS-C) is a relatively new and rare complication of COVID-19. This complication seems to develop after the infection rather than during the acute phase of COVID-19. This report aims to describe a case of MIS-C in an 8-year-old Thai boy who presented with unilateral lung consolidation. Unilateral whiteout lung is not a common pediatric chest radiograph finding in MIS-C, but this is attributed to severe acute respiratory failure.

**Case presentation:**

An 8-year-old boy presented with persistent fever for seven days, right cervical lymphadenopathy, and dyspnea for 12 h. The clinical and biochemical findings were compatible with MIS-C. Radiographic features included total opacity of the right lung and CT chest found consolidation and ground-glass opacities of the right lung. He was treated with intravenous immunoglobulin and methylprednisolone, and he dramatically responded to the treatment. He was discharged home in good condition after 8 days of treatment.

**Conclusion:**

Unilateral whiteout lung is not a common pediatric chest radiographic finding in MIS-C, but when it is encountered, a timely and accurate diagnosis is required to avoid delays and incorrect treatment. We describe a pediatric patient with unilateral lung consolidation from the inflammatory process.

## Introduction

Multisystem inflammatory syndrome in children (MIS-C) is a relatively new and rare complication of COVID-19. This complication seems to develop after the infection rather than during the acute phase of COVID-19. MIS-C affects children of all ages with the majority of patients ranging from 4 to 13 years old [1, 2]. Innate host immunity drives the multisystem hyperinflammation in MIS-C. Clinical features of MIS-C are signs of hyperinflammation and multi-organ dysfunction, with 51% developing myocarditis and cardiorespiratory failure [3]. Due to the recent emergence, the thoracic imaging findings of MIS-C are limited. Imaging features of MIS-C include pleural effusion, pulmonary edema, and acute respiratory distress syndrome, mostly involving both lungs [4]. In this report, we outline the case of a young boy, without an underlying condition, who presented with myocarditis, acute respiratory failure, and unilateral lung consolidation.

## Case presentation

A previously healthy 8-year-old boy was admitted to the hospital presenting with a high fever of 39.4 °C, an erythematous rash over the face and extremities, and decreased oral intake for one day. He had a history of a cold from COVID-19 infection 1 month ago and he never received the COVID-19 vaccination. On the third day of fever, he developed right cervical lymphadenopathy, conjunctival injection in both eyes, and a sore throat. He was admitted due to persistent high fever and poor intake at a private health care facility and was managed with symptomatic treatment. On the next day, he developed hypotension (84/56 mmHg) and respiratory failure (respiratory rate of 42 breaths/min and Spo_2_ of 85%). The patient received fluid boluses and endotracheal tube intubation. Then he was transferred to the pediatric intensive care unit (PICU) at our hospital.

**Fig. 1:**
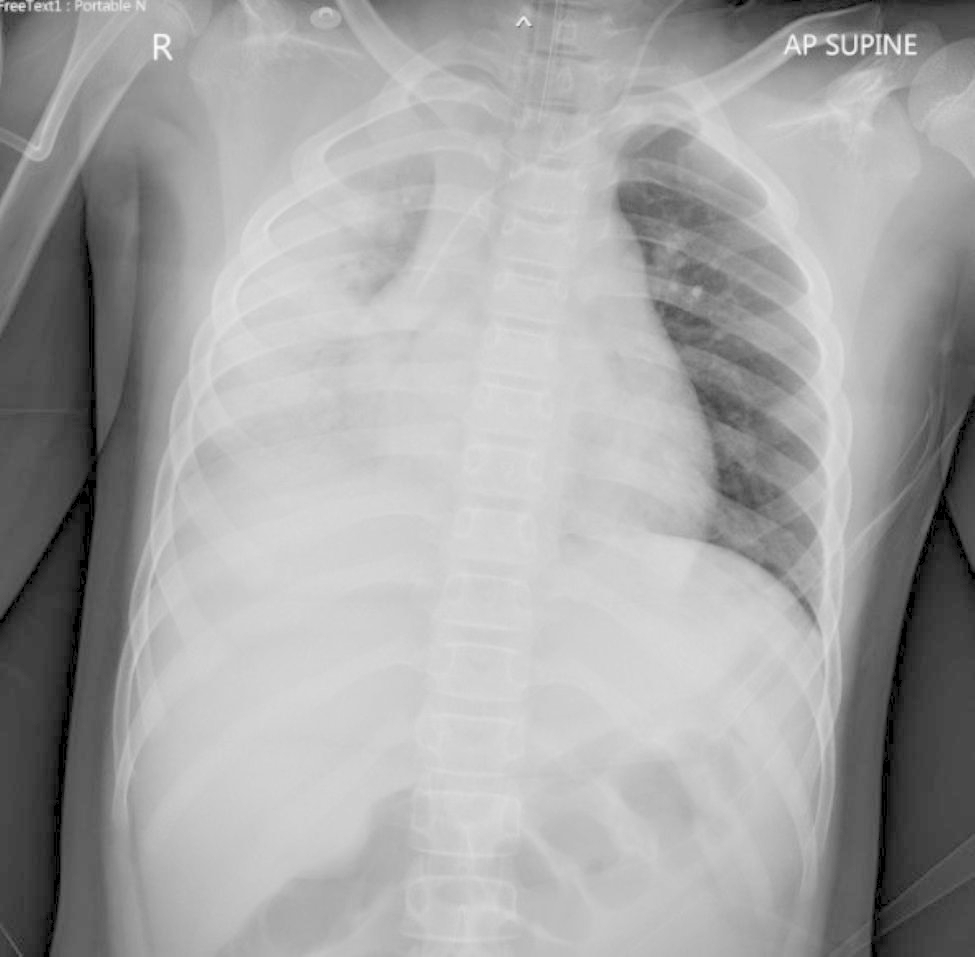
CXR showed patchy consolidation with an internal air bronchogram at the right lung

On admission, the patient was fully oriented, but restless. He exhibited tachypnea (RR 30–32/min). The mechanical ventilator settings were assisted control ventilation, pressure control mode, FiO_2_ 100%, pressure control of 30 cmH_2_O, respiratory rate of 22 times/min, and positive end-expiratory pressure of 9 cmH_2_O. After using the ventilator, an arterial blood gas study revealed a pH of 7.348, PO_2_ of 57 mmHg, PCO_2_ of 39.5 mmHg, HCO_3_^−^of 21.1 mmol/L, and lactate of 3.5 mmol/L. Chest radiograph showed patchy consolidation with an internal air bronchogram at the right lung with cardiomegaly (Fig. 1). Echocardiogram was performed and showed a prominence of the left anterior descending coronary artery; cross-sectional diameter of 4.3 mm with *z* score of 1.27 and perivascular brightness, decreased left ventricular (LV) function, and mild mitral valve insufficiency with an ejection fraction (EF) of 40%. There was no coronary aneurysm.

Laboratory studies showed no leukocytosis (white blood cell count: 6.07 *10^9^/L), and the white blood cell differential count showed 82.6% neutrophils and 14.5% lymphocytes. There were elevated blood levels for C-reactive protein (206.2 mg/L) and procalcitonin (17 ng/mL). Moreover, multiple clinical indexes exceeded the normal range, including alanine aminotransferase, aspartate aminotransferase, lactate dehydrogenase, and creatinine. Other laboratory results and trends are shown in Table 1. A nasopharyngeal swab sample tested for SARS-CoV-2 by real-time PCR was negative, but his serology tested positive for SARS-CoV-2-specific anti-SARS-CoV-2 nucleocapsid. Serum troponin T and troponin-I levels and N-terminal pro-B-type natriuretic peptide (NT-proBNP) were elevated, indicating cardiac ischemia or injury.

**Table 1:** Summary of laboratory results of the patient with MIS-C

Lab test	Admission	12 h	24 h	48 h	96 h	120 h	148 h	Reference
WBC	6.07	X	13.97	14.65	20.64	25.77	17.43	4–12 × 10^9^/L
Neutrophil	5.0	X	11.8	13.9	19.2	21.6	14.8	1.10–7.2 × 10^9^/L
Lymphocyte	0.9	X	1.8	0.6	0.8	2.3	1.7	1.30–7.20 × 10^9^/L
Hb	9.10	X	9.7	9.5	10	10.8	10.4	113–150 gm/L
Platelet	128	X	197	252	406	475	420	150–400 × 10^9^/L
CRP	206.2	218.32	175.79	74.77	45.17	29.89	16.16	≤ 1.20 mg/L
Procalcitonin	17	13.3	10.9	5.12	2.1	0.86	0.45	≤ 0.05 ug/L (ng/mL)
ESR	55	71	76	76	55	49	53	0–20
IL-6	152	16.9	11.5	3.7	2.6	2.2	X	0–7 pg/mL
Ferritin	522	X	X	X	X	X	X	4.6–204 ug/L
LDH	669	X	X	X	X	X	X	125–220 u/L
D-dimer	4697	X	X	X	X	X	X	0-550 ng/mlFEU
Fibrinogen	5.81	X	X	3.77	X	X	X	1.50–4.10 gm/L
Troponin T	99.59	X	30.72	26.03	25.10	X	X	≤ 15.6 ng/L
Troponin I	591	X	65.50	38.6	42.9	X	X	≤ 34 ng/L
AST	54	X	X	26	49	X	X	5–34 U/L
ALT	21	X	X	13	20	X	X	5–55 U/L
Albumin	23.3	X	19.7	19.3	23.4	X	X	35–50 g/L
NT-proBNP	16,645	X	6520	2385	3096	X	X	< 125 pg/mL

A diagnosis of MIS-C was made based on the above clinical features and paraclinical testing results. The patient remained hemodynamically unstable with features of shock and cardiac failure. Gentle fluid boluses with 0.9%NSS 10 ml/kg, broad-spectrum antibiotic (meropenem), norepinephrine (0.1 mcg/kg/min), and epinephrine (0.05 mcg/kg/min) were given.

**Fig. 2:**
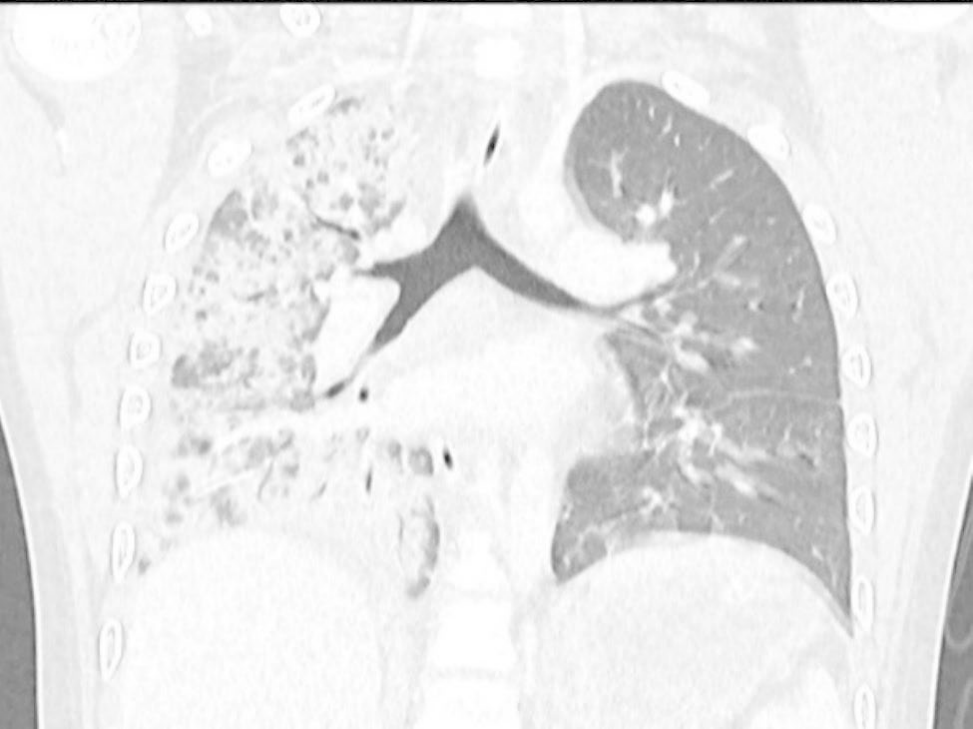
CT showed consolidation and ground-glass opacities in nearly the entire right lung

Intravenous immunoglobulin (IVIG; 1 g/kg/day) and methylprednisolone (2 mg/kg/day) were started. A chest computed tomography (CT) was performed which showed consolidation and ground-glass opacities of the right lung without evidence of pulmonary embolism (Fig. 2). Flexible bronchoscopy was performed and showed serous secretion with normal airway mucosa. Bronchoalveolar lavage fluid (BALF) was obtained. BALF cell populations were total of WBC 486 cells/mm^3^ (mononuclear cells 12%, neutrophils 88%), RBC 600 cells/mm^3,^ and no bacterial growth in BALF’s culture.

**Fig. 3:**
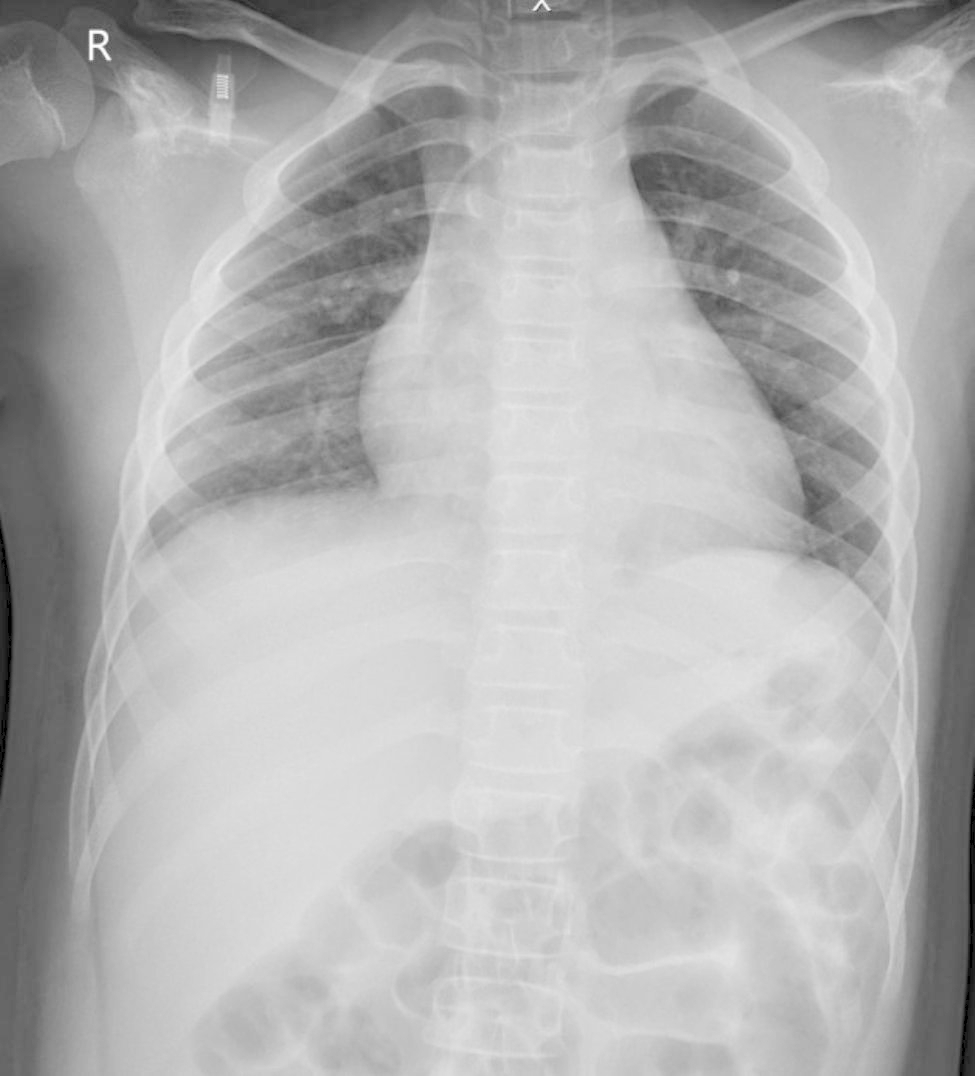
CXR showed resolution of patchy consolidation in the right lung

His fever had subsided dramatically after 4 h of treatment, and vasopressors could be discontinued after 12 h of treatment. IVIG was given for 2 days and aspirin was started. Methylprednisolone was given and tapered off in 2 weeks. Right lung patchy opacities were resolved with chest imaging on hospital day 3 (Fig. 3). Two sets of blood culture were negative. After 5 days of PICU admission, the patient was successfully liberated from a mechanical ventilator. LV dysfunction was reevaluated by an echocardiogram which revealed normal LV function without mitral valve insufficiency with an EF of 65%. On discharge, chest imaging was within normal limits. He was discharged home on day 8.

## Discussion and conclusions

This is a pediatric case of shock and multisystem inflammation associated with COVID-19. In our case, the patient met the five requirements’ criteria needed to make the diagnosis of MIS-C according to the Centers for Disease Control (CDC), five criteria including: (1) concurrent or previous (within the past 4 weeks) COVID-19 diagnosis by either PCR or antigen/antibody testing, (2) severe illness necessitating hospitalisation in those aged less than 21 years, (3) marked involvement or dysfunction of 2 organs or more involvement (cardiac, renal, respiratory, hematologic, gastrointestinal, dermatologic or neurological), (4) presenting fever ≥ 38^o^C for more than 24 h, and (5) exhibiting severe inflammation as per laboratory findings: elevated CRP, D-dimer, serum ferritin, erythrocyte sedimentation rate, fibrinogen, and interleukin-6 [5, 6].

MIS-C patients, Kawasaki disease (KD), and Toxic shock syndrome (TSS) share a number of overlapping clinical characteristics but numerous clinical and investigative results are different and aid in differentiating these conditions [7]. The median age of KD patients is often lower, mucocutaneous lesions and coronary artery anomalies are more common, and cardiac dysfunction and myocarditis are less common than MIS-C [8]. Toxic shock syndrome was more abrupt and advanced more quickly than in MIS-C. It was identified by multisystem involvement involving three organ systems, typically renal involvement. Often, toxic shock syndrome does not involve myocardial failure like MIS-C [7]. The presence of anti-SARS-CoV-2 IgM or IgG has been a good indicator to identify MIS-C.

The typical chest radiographic findings in MIS-C [5–9] are perihilar-peribronchial thickening, pleural effusions, bilateral multifocal ground-glass opacities and consolidative airspace opacities [10, 11]. Lung opacities were usually bilateral. Our patient’s chest radiograph and CT scan presented with asymmetrical rapidly progressive ground-glass and consolidative airspace opacities, mainly in the right lung, which is rare and unusual in the typical MIS-C. With a prior report from Winant AJ, et al., a radiograph of an 8-year-old girl who came with MIS-C, hypoxic respiratory failure, myocarditis, and acute kidney injury revealed an acute respiratory distress syndrome pattern with an asymmetric, right greater than left pattern [4]. Other differential diagnosis should be concerned especially bacterial pneumonia or complication of MIS-C such as pulmonary embolism pulmonary edema which can present with unilateral lung consolidation [12, 13]. In this case, his CXR returned to normal in 3 days after treatment with anti-inflammatory medication along with no evidence of infection. Hyperinflammatory process [14] could be the cause of his unilateral lung consolidation. Furthermore, rapid recovery of left ventricular systolic dysfunction in this patient suggests that his dysfunction might be from transient myocardial stunning [15] secondary to systemic inflammation [16]. A series of imaging helped us to assess the patient’s condition and guided the diagnosis.

Our patient had experienced MIS-C associated with shock, myocardial injury, and acute respiratory failure, including the need for a mechanical ventilator, fluid resuscitation, and inotropic agents due to circulatory failure. The patient’s symptoms were successfully treated with intravenous immunoglobulins (IVIG) and glucocorticoids, and he was discharged with a resolution of his symptoms. Regarding the variability in treatments, Joseph Y. Abrams et al., reviewed 4,901 patients with MIS-C. They reported that 86% of patients were given IVIG, 78% given steroids, 21% given immunomodulators (tocilizumab, anakinra, cyclophosphamide, rituximab), 73% given aspirin, 44% anticoagulation, 38% required vasopressors and 1.5% required ECMO [16]. Despite the variations in therapy, a recent literature review of the MIS-C treatment options concluded that there is no proof that IVIG alone, IVIG combined with steroids, or IVIG combined with immunomodulators results in greater rates of recovery [17].

This case highlights the need for a greater understanding of cases with severe MIS-C. There has not yet been a single MIS-C presentation that applies to all phenotypes because knowledge of the variety of phenotypes is still developing. These findings may not be generalisable to a child population and there is very little in the literature regarding unilateral ground-glass opacities and radiologic findings in MIS-C, but it gives insight into the challenges of diagnosis and choosing a treatment modality.

## Conclusions

Unilateral whiteout lung is not a common pediatric chest radiographic finding in MIS-C, but when it is encountered, a timely and accurate diagnosis is required to avoid delays and incorrect treatment. This case highlights pediatric patients with unilateral lung consolidation from the inflammation process from MIS-C.

## Data Availability

All data and materials are available upon reasonable request from Tidarat Sriboonyong.

## Abbreviations

ALT: alanine aminotransferase
AST: aspartate aminotransferase
CK-MB: creatine kinase-MB
Cr: creatinine
CRP: C-reactive protein
ECMO: Extracorporeal membrane oxygenation
IVIG: intravenous immunoglobulins
LDH: lactate dehydrogenase
MIS-C: Multisystem inflammatory syndrome in children
NT-proBNP: N-terminal pro-B type natriuretic peptide
TnT: Troponin
WBC: white blood cell
